# A review of level-1 visual perspective-taking: potential relationship with the uncanny valley effect

**DOI:** 10.3389/fpsyg.2024.1394744

**Published:** 2024-11-12

**Authors:** Cong Fan, Weiqi He

**Affiliations:** ^1^Research Center of Brain and Cognitive Neuroscience, Liaoning Normal University, Dalian, China; ^2^Key Laboratory of Brain and Cognitive Neuroscience, Dalian, Liaoning, China

**Keywords:** level-1 visual perspective-taking, implicit mentalizing, submentalizing, debate, uncanny valley

## Abstract

Calculating others' visual perspective automatically is a pivotal ability in human social communications. In the dot-perspective task, the ability is shown as a consistency effect: adults respond more slowly to judge the number of discs that they can see when a computer-generated avatar sees fewer discs. The implicit mentalizing account attributes the effect to relatively automatic tracking of others' visual perspective. However, the submentalizing account attributes the effect to domain-general attentional orienting. Accordingly, the current study focuses on elucidating the ongoing implicit mentalizing vs. submentalizing debate. The review tried to shed light on the debate regarding level-1 visual perspective taking and its potential relationship between the uncanny valley effect. Future research may focus on new manipulations of uncanny valley effect to further uncover the relationship between uncanny valley effect and level-1 visual perspective taking. This may provide new insight into the debate and the processing mechanisms of level-1 visual perspective-taking and uncanny valley effect, which may be beneficial for AI development.

## 1 Introduction to the review

Theory-of-mind (ToM) refers to the ability to reason about how individuals' mental states (beliefs, intentions, emotions, etc.) influence their behaviors (Low et al., 2016; Rakoczy, 2022; Singer, 2006; Wellman et al., 2001), which plays an important role in human social interactions. ToM researchers have divided the processing of understanding others' visual perspectives into two broad levels: Level 1 requires judgments about what someone else sees (known as level-1 visual perspective taking, L1VPT); Level 2 requires attributions about how different individuals can have different interpretations of the same stimulus depending on their viewing circumstances (known as level-2 visual perspective-taking, L2VPT). As the basis of later ToM processing, L1VPT has been attracting major research attention from scientists in diverse areas that include animal behavior, ecology and human ToM cognition and development, ever since Premack and Woodruff (1978) raised the possibility that certain abilities to track others' viewpoints may be evolutionarily ancient and form the basis for the development of more complex mental-state reasoning. For the review, I shall summarize studies regarding L1VPT, uncanny valley and the uncanny valley effect on L1VPT. And such studies may especially timely provide new evidence for the contradictions and controversies on the extent to which adult humans automatically encode what is seen when someone gazes.

## 2 Previous work—L1VPT

### 2.1 Altercentric intrusion in L1VPT

Even though human beings' tendency to be egocentric highlights that some ToM processes can be cognitively effortful, there is evidence showing that people can easily and effortlessly compute others' visual perspectives (De Lillo and Ferguson, 2023). For instance, Sodian et al. (2007) tracked infants' eye movements to find it was easy to understand another person's discrepant visual experience. Specifically, 14-month-old infants looked longer at an actress's goal-directed action for a novel object when the old target was visible than when the old target was invisible to her (but still visible to the infants). The looking behaviors were evoked under the circumstance of passively picture-viewing without any other task instruction. Thus, their looking time patterns suggest that 14-month-old infants can easily compute adults' visual perspectives independently of their own perspectives (i.e., L1VPT ability). The findings fit with the speculation that perspective computation reflects infants' apparently sophisticated ToM as indirectly measured by their looking time responses (e.g., Baillargeon et al., 2010) (nonetheless, it is important to acknowledge that infants' success on non-verbal tasks are subject to replication problems, and their success can also be explained by a range of sub-mentalistic processes) (Ruffman et al., 2012; Zaadnoordijk et al., 2022).

Similar to infants' visual computation, adults can effortlessly track others' visual perspectives (Samson et al., 2010), which was reflected by the finding that adults were slower and less accurate to judge the number of dots they saw when an avatar saw a different number of dots (i.e., consistency effect) on self-perspective trials. The consistency effect elicited without explicit judgement about the avatar's visual perspective was interpreted as an effect of altercentric intrusion. That is, adults' computation of the avatar's visual perspective was task-irrelevant and yet appeared to be undertaken in a way that interfered with judgements on their own perspectives. Additionally, effortless calculation of others' visual perspectives in L1VPT processing was found to be independent of executive-function resources (Qureshi et al., 2010).

### 2.2 The dot-perspective paradigm and generalization of consistency effect

#### 2.2.1 The dot-perspective paradigm

The dot-perspective paradigm was created by Samson et al. (2010) as an experimental paradigm for measuring L1VPT processing. The essential idea behind the task is that if adults can implicitly track another person's visual perspective, then participants should do so even when they do not need to. In the paradigm, disc(s) were presented on the left- or right-side wall of a room with a computer-generated human avatar standing in the center of the room and facing to one side of the walls. Two kinds of visual perspectives—“You see N” on “Self-perspective” trials (“N” ranges from 0 to 3 dots), “S/he sees N” on “Other-perspective” trials—were presented before the scene. The participants were required to judge whether the picture matched the given perspective, leading to matching and mismatching trials. On half of the trials (Congruent condition), the avatar and the participant could see the same disc(s). On the remaining trials (Incongruent condition), the participant could see the disc(s) that were invisible to the avatar.

Consistent with previous studies of egocentric biases (i.e., strong biases toward the participants' own perspectives on other-perspective judgements) (e.g., Birch and Bloom, 2007), findings indicated egocentric intrusions when the participants were instructed to take the avatar's visual perspective. For instance, Samson et al. (2010) found slower response times and lower accuracy in incongruent condition compared to congruent condition (i.e., Consistency effect) on other-perspective trials. Additionally, the novel and key finding of the study was that the participants made more errors and responded more slowly in inconsistent condition compared to consistent ones when making self-perspective judgements. The finding suggested that adults could rapidly and effortlessly take the avatar's visual perspective even when not required to do so. The researchers explained the result as an effect of altercentric interference/intrusion, namely, the participant's own visual perspective was interfered by the implicit computation of the avatar's visual perspective that was task-irrelevant (Samson et al., 2010).

#### 2.2.2 Generalization of the self-perspective-related consistency effect

The consistency effect on self-perspective trials, considered as an effect of altercentric intrusion in L1VPT processing, has been replicated and extended. Further, the effect has even persisted under secondary task conditions where cognitive-resource tasks are added. In the original study, Samson et al. (2010) firstly observed that adults performed more slowly and made more errors on inconsistent trials compared to consistent trials when they were asked to take their own perspectives (i.e., the consistency effect on self-perspective trials). Later, a line of L1VPT-related experiments using the dot-perspective task has generalized the consistency effect under self-judgement circumstances. In addition to Surtees et al.'s (2016) replication of Samson et al.'s (2010) findings, Surtees and Apperly (2012) extended the altercentric intrusion effect from adults to 6–10-years-old children. More importantly, the effect persisted when considering its relationship with cognitive load. The consistency effects remained when participants judged their own perspectives regardless of time pressure for responses (i.e., shorter-deadline of 600 ms compared with a long-deadline of 1,200 ms) (Todd et al., 2017), and also persisted when the dot-perspective task was performed together with a secondary task requiring executive-function resources (Qureshi et al., 2010). The two studies, then, demonstrated that tasks requiring cognitive resources did not influence the elicitation of efficient computation of others' visual perspectives on self-perspective judgements.

### 2.3 The mentalizing vs. submentalizing debate

#### 2.3.1 The content of the debate

Recent work has suggested that the processing of others' minds depends on two cognitive systems. One is a flexible system that enables us to explicitly reason about how others' mental states (beliefs, intentions, emotions, etc.) influence their behaviors. The other is an efficient system that allows us to automatically track others' mental states (Kovács et al., 2010; Low et al., 2016; Schneider et al., 2014). Samson et al. (2010) suggested that the efficient system enables us to automatically track what someone else sees (i.e., L1VPT). However, there is obvious debate over whether and to what extent the efficient system is specialized (implicit and automatic mentalizing account: e.g., Apperly, 2010) or domain-general (submentalizing account, e.g., Heyes, 2014). Specifically, the debate was sparked by the adults' performance in the dot-perspective task of L1VPT. That is, implicit mentalizing account claims that the consistency effect on self-perspective trials is elicited by implicit and effortless computation of the avatar's visual perspective via connecting her/his line-of-sight with the discs. The submentalizing account, alternatively, holds the view that the effect is evoked by merely attentional orienting produced by the directional but not agentive features of the avatar (e.g., head and/or body directions).

#### 2.3.2 Implicit mentalizing account

Researchers supporting the implicit mentalizing account claim that the consistency effect on the self-perspective judgement is invoked by implicit computation of others' mental state of seeing. Specifically, if participants could easily understand others' visual information, then they would rapidly and efficiently track the avatar's visual perspective in the dot-perspective task even without the explicit judgements of others' perspectives.

With respect to the implicit mentalizing account, researchers regard eye gaze as the key factor, which can be supported by the evidence showing the important role of eye gaze in mentalizing-related processes. For example, Baron-Cohen et al. (1995) observed that 3 and 4-year-old children can infer a person's mental state of wanting a chocolate bar (i.e., desires) by tracking others' eye-gaze direction to the target. More importantly, some researchers have found a strong relationship between the eye gaze and L1VPT processing (Sodian et al., 2007). They reported that 14-month-old infants can make expectations about an agent's goal-directed action based on understanding whether or not the line of sight between the agent's eyes and an object is physically unblocked (i.e., L1VPT ability). Based on these studies, eye gaze can convey information about others' visual perspectives. Therefore, manipulation of visual access has been created to measure L1VPT processing tapped into the mentalizing-related process.

Several studies have manipulated the gazer's line-of-sight as a novel way to clarify the mentalizing vs. submentalizing debate. Among those studies, only one has found evidence for implicit mentalizing (Furlanetto et al., 2016). Specifically, Furlanetto et al. (2016) manipulated the avatar's visibility to explore the debate by adopting transparent goggles (i.e., visible condition) and opaque goggles (i.e., invisible condition). The participants were checked to be able to associate different colored goggles with the corresponding avatar's ability to see (i.e., in the seeing condition, the red goggles worn by the avatar were transparent, whereas in the non-seeing condition, the orange goggles worn by the avatar were opaque). The participants were instructed to judge their own perspectives or perspectives of the avatar wearing the different colored goggles. The authors found that participants judged their own perspectives more slowly and less accurately in the inconsistent condition compared with the consistent condition, but the consistency effect was present in the seeing but not non-seeing conditions. The explanation of the discrepancy was that participants had different beliefs of the avatar's epistemic state of seeing via understanding the transparent and opaque features of the goggles, which then lead to the connection of the gazer's line-of-sight with the disc(s) on the wall(s) in the seeing condition but the disconnection in the non-seeing condition.

The findings of this study cast doubt on the submentalizing account claiming the role of directional information as it would predict the consistency effects in both visible and invisible conditions due to the identical directional features. Thus, participants can implicitly and efficiently compute the visual perspective of the avatar wearing transparent goggles even when they were not required to do so, which lent support to the implicit mentalizing account. However, the study is limited on its own. Specifically, the study cannot rule out the carry-over effect between self- and other-perspective conditions as the two conditions presented in the intermixed block. Therefore, the consistency effect on self-perspective judgements may be contaminated by explicit judgements about others' perspectives. To explore whether L1VPT processing is implicit mentalizing, it would be better to separate seeing condition from non-seeing condition.

#### 2.3.3 The submentalizing account

Researchers supporting the submentalizing account have claimed that the consistency effect is elicited by domain-general mechanisms that are not specialized for processing of others' minds (e.g., attentional orienting, Heyes, 2014). Specifically, it is the directional features of the avatar that modulate participants' attentional shifts toward the number of dots on one side of the room. Therefore, on consistent trials of the dot-perspective task, the directional property of the centrally presented avatar oriented participants' attention toward the dot(s) on the target wall; whereas on inconsistent trials of the dot-perspective task, the directional property of the avatar oriented participants' attention neither to the dot(s) on the target wall nor to all the dots on both targeted walls. Then, the directional property of the centrally presented avatar may trigger more errors and slower response times in the inconsistent condition compared to the consistent condition. Related studies that tried to cast light on the debate by adding arrows as control stimuli relative to avatars and by manipulating the agent's line of sight via opaque barriers will be described in the next two subsections.

##### 2.3.3.1 Submentalizing and arrows

Advocates of the submentalizing account have cited literature showing that in addition to social stimuli, semi-social and/or non-social stimuli can also generate self-consistency effects (Nielsen et al., 2015; Santiesteban et al., 2014; Todd et al., 2017; Wilson et al., 2017). For example, Santiesteban et al. (2014) modified the dot-perspective task by adding new trials where the avatar was replaced with an arrow with similar low-level directional features (e.g., height and position). Self-consistency effects of comparable size were found in the avatar and arrow conditions, suggesting that the consistency effect in the dot perspective task may be triggered by domain-general processes such as attentional orienting. Furthermore, attentional-orienting mechanism of L1VPT processing is also reflected by the findings of significant consistency effects in the dot-perspective task regardless of the sociality of the centrally presented stimuli [i.e., all the consistency effects are significant but different in magnitude: directional avatars (social stimuli) > directional arrows (semi-social stimuli) > directional, dual-colored blocks (nonsocial stimuli)] (Nielsen et al., 2015). Even though arrows have been found to be able to trigger self-consistency effect as avatars could, there are limitations with such approaches (Nielsen et al., 2015; Santiesteban et al., 2014; Todd et al., 2017; Wilson et al., 2017). First, participants' expertise with arrows (from previous experiences of being exposed to arrows) may make them treat arrows as purposefully designed (by the experimenters) to prioritize some perspective on the scene that may be similar to the avatar-triggered L1VPT. Furthermore, whilst the arrow that Santiesteban et al. created has the directional property, it also potentially has animacy because its height, shape, color distribution and area were matched to the avatar [also in Experiment 1 of Conway et al.'s (2017) study]. Indeed, studies show that adults may attribute mental states to simple geometric shapes (e.g., Surian and Geraci, 2012), suggesting that, rather than being submentalisers, adults may be supermentalisers. Thus, it is not clear that these findings rule out the implicit mentalizing account for automatic L1VPT.

##### 2.3.3.2 Submentalizing and visual barriers

In addition to comparing avatar- and arrow-related self-consistency effects in the dot-perspective task, other researchers attempted to manipulate the gazer's visibility by using barriers to explore the mentalizing vs. submentalizing debate (Cole et al., 2016; Conway et al., 2017; Langton, 2018; Wilson et al., 2017). Failing to replicate Furlanetto et al.'s (2016) findings, these studies have found that the self-perspective-related consistency effect even persists when agents' “non-seeing” conditions are imposed by using barriers. The lack of difference between the visible and invisible conditions demonstrates that directional information of the avatar instead of mentalistic processing of seeing elicited the consistency effect, supporting the submentalizing account.

To render discs in the dot-perspective paradigm visible and invisible, Conway et al. (2017) manipulated an avatar's visual access by using a cloaking device or goggles that were worn by the avatar. In Experiment 1, the authors adopted visible and invisible telescopes within a cloaking device to render the seeing condition and non-seeing condition, respectively. In Experiments 2 and 3, the avatar wearing a goggle with a transparent internal lens could see whereas the avatar wearing a goggle with opaque internal lens (i.e., the lens were covered by a blackout material) could not. Inconsistent with Furlanetto et al.'s (2016) findings, they found the consistency effects in both visible and invisible conditions even though they had ruled out the carry-over effect by intermixing self- and other-perspectives. It may be because certain barriers used in the study for conveying non-seeing states can be relatively complex (e.g., subtly colored goggles, and their unique cloaking properties). Additionally, it may be relatively hard for participants to regard the barrier scenario as the non-seeing condition, especially when they only had a limited time-period to grasp the novel scenario. To address the potential issue of the aforementioned barriers, Wilson et al. (2017) employed easily-recognizable blindfolds to render the discs invisible. However, the following points may be regarded as being potential interpretations for the finding of the consistency effect in the non-seeing condition. Self- and other-perspective trials were intermixed, and the alternate presentation of these two types of trials may lead to a carry-over effect. Consequently, participants may be explicitly tracking the avatar's visual perspective even though no related instruction was displayed. Furthermore, these barriers may not evoke effective non-seeing scenarios as they occupied a relatively small part of the avatar.

Instead of using relatively small eyes-covered devices, Langton (2018) displayed a pair of big opaque boards between the gazer and the target discs to create the invisible scene and, additionally, replaced computer-generated avatars with photographs of real humans (Experiment 1) or with a gazer sitting face-to-face with participants (Experiment 2). The findings of both experiments spoke against implicit mentalizing but supported the submentalizing accounts by observing a significant consistency effect in the invisible condition of the dot-perspective task. However, the study also had the following limitations. First, the barrier manipulation in Experiment 1 may not have effectively created a non-seeing scenario. Specifically, in non-seeing condition, the lengthy distance between the centrally presented gazer and the peripherally presented barrier could have led participants to perceive that the target discs still fell within the gazer's visual field, particularly under a limited response duration (2 s). Second, the revised dot-perspective task in Experiment 2 was distinct from the classic Samson et al.'s (2010) task. Specifically, an arrow cue appeared behind the participant's head instructed the gazer to turn his head toward one of the two laterally presented monitors. Then, 2 s followed by the presentation of the arrow, the targeted discs were displayed on one or two lateral monitor(s). In the situation, the head turn may be accomplished before the appearance of the target discs, which may trigger an SOA variable. Importantly, the “SOA” factor made the classic dot-perspective task similar to the stimulus-presentation mode of the classic Posner task (i.e., a well-known task tapping attentional orienting), which then, may trigger attentional orienting effect instead of visual-perspective-taking-related processing.

#### 2.3.4 Dissociating the mentalizing from the submentalizing accounts

Some researchers have attempted to dissociate the mentalizing account from the submentalizing account by contrasting relevant paradigms. Gardner et al. (2018) attempted to dissociate the competing accounts by contrasting effects in modified dot-perspective task with the Posner task. The Posner task has been widely used to measure attentional orienting, in which a cue is first presented, followed by a target with a stimulus onset asynchrony (SOA), and then, participants are required to detect the target location. In Experiment 1, Gardner et al. examined if reflexive attention orienting can sufficiently induce the self-perspective-related consistency effect that would be considered as automatic visual perspective-taking in the dot-perspective task. They adopted a revised dot-perspective task by eliminating the “YOU perspective” instruction from the original Samson et al. (2010) study. The novel dot-perspective task made the participants unaware that they were completing a perspective-taking task. Thus, in Experiment 1, removal of the “YOU perspective” instruction resulted in a non-significant consistency effect, demonstrating that the effect cannot be evoked merely by reflexive attention orienting. In Experiment 2, they used the Posner task with dot-perspective-task's stimuli to investigate whether the attentional orienting property of the avatar contributed to the consistency effect that was previously induced in the dot-perspective task. They found the cue-validity effect only for longer SOAs. Specifically, adults were faster to detect a target when the avatar was directed to the target (valid trials) compared to when the avatar was directed away from the target (invalid trials) when SOA was 600 ms but not 100 ms or 300 ms. The findings demonstrated that voluntary rather than reflexive attention shift contributed to the consistency effect in the dot-perspective task. Taken together, the consistency effect in the classic dot-perspective task might be less automatic than first reported. Nonetheless, the discrepancy between visual-perspective-taking and attention-shift processes cannot be directly distinguished.

Previous findings revealed that compared to stance-maintained avatars (i.e., avatar's head and torso faced to the same wall), stance-averted avatars (i.e., avatar's head was oriented to one wall whereas the torso faced to the participant) induced an increased attentional orienting effect (e.g., Hietanen, 2002). Accordingly, Gardner et al. (2018) hypothesized that avatar-stance may modulate attentional orienting but not visual perspective-taking. They attempted to distinguish the implicit mentalizing from the submentalizing accounts by manipulating avatar stance (stance-averted vs. stance-maintained). Specifically, they explored whether avatar-stance could differently modulate the effect from visual-perspective-taking tasks (i.e., consistency effect in the dot-perspective task) and from attentional-orienting tasks (i.e., cue-validity effect in Posner task). Experiment 1 used the Posner cueing task to examine the cue-validity effect, finding that the target was more slowly to be detected in the invalid condition compared to the valid condition. The attentional orienting effect was modulated by avatar stance, which is reflected by the significant effect for stance-averted rather than for stance-maintained avatars. Experiment 2 adopted the dot-perspective task to replicate the classic consistency effect. More importantly, avatar-stance did not moderate the magnitude of the consistency effect in the classic visual-perspective-taking task. Accordingly, the dissociation between attentional orienting and visual-perspective-taking processes casts doubt on the submentalizing hypothesis regarding the role of the directional cue but supports the implicit mentalizing hypothesis.

#### 2.3.5 The implications of investigating the debate

The implicit mentalizing vs. submentalizing debate has important methodological and theoretical implications. In the methodological aspect, the debate challenges the effectiveness of the dot-perspective paradigm as a measure of L1VPT ability. It is important to find a universally-recognized way to measure and clarify L1VPT processing, which can lay a foundation for connecting L1VPT with the later more complex ToM processes and understanding the related social communications. Theoretically, the debate raises the question about the efficient part of ToM processing system (i.e., whether people can effortlessly track others' mental states, e.g., Meert et al., 2017). Practically, resolution of the debate is important because it is beneficial to further, understand related dysfunction in social behaviors in atypical individuals. For example, psychopathic patients have been found to have deficits in L1VPT ability, and their dysfunction in effortlessly taking others' visual perspective have been demonstrated to be correlated with their callous and criminal behaviors in real-world (Drayton et al., 2018; Baskin-Sommers and Brazil, 2022). Furthermore, a recent review (Capozzi and Ristic, 2020) has demonstrated social orienting attribute to the integration of the attribution of mental states and the manipulation of domain-general attentional processes. However, whether it is the case needs further investigations.

## 3 Previous work—Uncanny valley

The uncanny valley (UV) refers to the phenomenon that as an agent approaches human likeness, there is a sudden dip in our affinity for it, and negative emotions such as feelings of eeriness and disgust are triggered when we human beings are confronted by an artificial being (e.g., avatars and robots) who looks and/or acts like humans, but is not quite lifelike enough (Mori, 1970; Mori et al., 2012; Yam et al., 2021). At an early stage, Mori (1970) proposed that any type of human-likeness manipulation could result in a characteristic UV curve for affinity (i.e., Naïve Hypotheses); that more negative affinity (i.e., lower perceptual familiarity and/or more negative emotional valence) might be generated by morbid characters (e.g., corpses or zombies) when compared to any other characters (i.e., Morbidity Hypothesis); and that more affinity may be evoked by moving characters than still ones (i.e., Movement Hypotheses) (Kätsyri et al., 2015). Afterwards, there are theories that can interpret the UV effect from a cognitive perceptual, or evolutionary perspective (Di Natale et al., 2023).

### 3.1 Cognitive theories on UV effect

#### 3.1.1 Category uncertainty theory

In addition to Mori's original hypotheses about the UV effect, there are several potential explanations for the UV effect from a cognitive mechanism perspective. One view suggests that category uncertainty during identification results in the UV effect (Jentsch, 1906/1997; MacDorman and Ishiguro, 2006; Pollick, 2010). The idea is that when presented with stimuli sharing human-like features, participants will attempt to classify the stimuli into human and non-human, or inanimate and animate categories; and observers' lack of certainty about what an entity is (e.g., entities that are positioned at the category boundary), is speculated to give rise to stronger negative affective response (MacDorman and Ishiguro, 2006; Pollick, 2010; Kätsyri et al., 2015). Moreover, based on Cheetham et al.'s (2014) claims, Kätsyri et al. (2015) proposed that within a categorical perception framework, the UV hypotheses suggested that increased perceptual discrimination challenges for neighboring character pairings would be linked to greater negative emotional responses. However, increasing an entity's overall category uncertainty does not heighten cold, eerie feelings. In fact, MacDorman and Chattopadhyay (2016) found that in a categorization task on animacy (living vs. inanimate) and realism (computer animated vs. real) indices, the eeriest and coldest stimuli were those categorized with the most certainty. Despite that the fact category uncertainty theory has been criticized, there are a wide range of theories proposed for the UV, many of which have not received as much negative criticism as category uncertainty.

#### 3.1.2 Mind perception and expectation violation theories

According to mind perception hypothesis, humanoid robots can be eerie as their realism leads people to ascribe to them the abilities of feelings and sensations. Yet, these abilities are unlikely to develop in robots (Gray and Wegner, 2012). Expectation violation theory expands the mind perception hypothesis by positing that people expect humanoid robots with human-like appearances to behave like humans (i.e., human-directed expectations). However, the humanoid robots frequently violate the above expectations by, for instance, moving mechanically (Wang et al., 2015), resulting in negative emotional responses and feelings of eeriness and coldness (Broadbent, 2017). Furthermore, Saygin et al. (2012) interpreted expectation violation theory as prediction errors for viewed actions in predictive coding that may be triggered by a real human moving like robots (e.g., a performance artist painted himself/herself gold, stood in front of a cathedral and moved like a robot) (Saygin et al., 2012).

### 3.2 Perceptual theories on UV effect

#### 3.2.1 Perceptual mismatch theories

Apart from some cognitive theories regarding the UV effect, several theories can explain the UV effect from a perceptual viewpoint (i.e., the UV effect is regarded as a stimulus-driven effect). Moore (2012) explains the perceptual mismatch hypothesis from a mathematical perspective, namely, perceptual distortion caused by conflicting cues supplied by a Bayesian model may result in negative, fearful or even violent responses (Moore, 2012). Moreover, an explanation based on realism inconsistency between the human-likeness levels of specific sensory cues would suggest that, high skin realism, for instance, elicits neurocognitive expectancies of high eye realism (but not vice versa), and inconsistencies in realism could violate neurocognitive expectancies, leading to large feedback error signals (MacDorman and Chattopadhyay, 2016; Saygin et al., 2012). More studies are reporting that uncanniness can be explained by realism inconsistency theory resulting from mismatches in perceptual processing of the human likeness of an entity's features at different levels (e.g., when human face and skin paired with computer-generated eyes; human voice with robot head) (e.g., Chattopadhyay and MacDorman, 2016; Di Natale et al., 2023; MacDorman and Chattopadhyay, 2017; Zhang et al., 2020). Research overall suggests that realism inconsistency theory in contrast to category uncertainty theory is a better explanation for the UV phenomenon. Apart from realism inconsistency theory, which is a form of perceptual mismatch hypothesis, the perceptual mismatch hypotheses also involve sensitivity to atypical features (e.g., human-like entities with atypical features will generate a stronger negative reaction when compared to artificial entities with atypical features, as well as human-like and artificial entities with typical features; Kätsyri et al., 2015), as evidenced by findings indicating that the most negative affinity was triggered when artificially enlarged eyes were matched with real human faces (Seyama and Nagayama, 2007).

#### 3.2.2 Configural processing theories

Configural processing theories assume that deviations in the configural pattern of specific and familiar stimuli cause the UV effect (Kätsyri, 2018; Kätsyri et al., 2019). The sensitivity to facial proportions heightens as human likeness increases, and a feeling of eeriness is generated by deviation from the ideal proportions of more attractive faces (Green et al., 2008). Furthermore, the Thatcher illusion also supports configural processing theories' explanations of the UV effect. Specifically, a face with inverted eyes and mouth can make the appearance grotesque, which indicates that recognizing deviations in configural processing can elicit uncomfortable reactions (Diel and MacDorman, 2021). Recently, Diel and Lewis's (2022) configural processing account interprets the UV effect by a moderated linear function for which perceptual specialization enhances the sensitivity to deviating stimuli (e.g., voice distortions), which gives rise to an increase in uncanniness (Diel and Lewis, 2024).

### 3.3 Evolutionary theories on UV effect

#### 3.3.1 Threat avoidance theory

Mori (1970) initially proposed the importance of the feeling of uncanniness in human self-preservation. Diseases and death have been two primary threats to human evolution. Avoidance of threats (e.g., pathogens or unit mates, Moosa and Ud-Dean, 2010) can explain the UV effect. One viewpoint, known as the Pathogen Avoidance hypothesis, holds that the UV effect is associated with an evolutionary mechanism for pathogen avoidance. Specifically, people connect the humanoid robots' (with great human-likeness) flaws with indicators of transmissible diseases, causing disgust (Wang et al., 2015). Even though previous work provides indirect evidence for the Pathogen Avoidance hypothesis by showing the correlation between disgust and UV (MacDorman and Entezari, 2015; Villacampa et al., 2019), direct support for this hypothesis is lacking. Another explanation regarding threat avoidance is the Mortality Salience hypothesis, which posits that the defects of human-like entities can remind people of death and the entities may be regarded as dead persons who come alive; that the feeling of uncanniness is elicited since defense systems are activated to deal with the fear of death and anxiety for mortality (MacDorman and Ishiguro, 2006). Moreover, the Mortality Salience hypothesis is supported by the finding of the association between the physical body's vulnerability and impermanence sensitivity and the android's eerie evaluations (MacDorman and Entezari, 2015).

#### 3.3.2 Psychopathy avoidance theory

Psychopathy avoidance Theory links uncanniness with psychopathic personality traits. Tinwell et al. (2013) found that virtual characters lacking upper facial expressions were perceived to be most uncanny, and this uncanniess could be strongly predicted by psychopathy assessments (Tinwell et al., 2013). The researchers believe that the virtual characters without upper facial expressions may be uncoordinated with other facial movements, failing to express their emotions as well as potentially implying an effort to conceal their negative personality traits. Additionally, the personality traits may be connected with psychopathy with other aspects (e.g., jerky movement, MacDorman et al., 2010; lip-sync error, Tinwell et al., 2010), resulting in the feeling of uncanniness. Therefore, the uncanniness may be a trigger of avoidance response.

### 3.4 Dehumanization theory

The UV effect can be interpreted from cognitive, perceptual and evolutionary perspectives; nonetheless, these viewpoints overlook to confirm the underpinning prediction that individuals may spontaneously recognize a human-like entity as a person (Wang et al., 2015). However, the Dehumanization Theory may address the issue. Dehumanization is known as the capability to regard an individual or group as lacking humanness—the characteristics that characterize what it means to be human (Haslam and Loughnan, 2014). There are two forms of dehumanization: “animalistic dehumanization” and “mechanistic dehumanization”. Animalistic dehumanization refers to a process where the denial to others of human unique characteristics makes entities animalistic (i.e., lack of civility, refinement, intelligence and self-control), whereas mechanistic dehumanization refers to a process where the denial to others of human nature makes entities mechanistic (i.e., lack of emotion, warmth and individuality) (Christoff, 2014; Haslam, 2006). The dehumanization hypothesis is compatible with previous hypotheses by proposing that the more human-like features are attributed to human replicas such as androids, the more probable it is that recognizing their mechanistic characteristics elicits the dehumanization process, resulting in a sense of uncanniness (Wang et al., 2015).

## 4 Previous work—The relationship between L1VPT and UV

To date, only two studies have involved the effect of uncanny valley on L1VPT. MacDorman et al. (2013) have investigated the extent to which automatic visual perspective-taking may be impaired by eerily realistic stimuli on the dot perspective task. In Experiments 1 and 2, the investigated performance on the dot perspective task when the stimulus was an inanimate object (e.g., an arrow or a chair), a robot (e.g., R2D2 from Star Wars), a fantasy being (e.g., a zombie), a non-human animal (e.g., a bee) and a human being (e.g., a male avatar). There was no difference in the altercentric interference effect whether the character in the dot perspective task displayed high human photorealism (the human avatar) or low human photorealism (inanimate objects). There was also no difference in the altercentric interference effect whether adults rated the character as displaying low eeriness (inanimate objects), moderate eeriness (robots, non-human animals and humans) or high eeriness (fantasy beings). The main finding of Experiment 1 was that participants were unable to ignore the irrelevant perspective (i.e., showed slower RTs on the self-inconsistent trials), irrespective of the human photorealism or eeriness of the stimuli. The main effect was upheld in Experiment 2 when self-trials and other-trials were blocked. Experiments 1 and 2, however, suffer from methodological inadequacies. First, different quantities of directional cues were given by different characters (e.g., body orientation was a directional cue by the human avatar, but body as well as arm orientation were directional cues by the zombie character). Second, physical properties (e.g., color, shape, angle of bodily stance) between the different entities were all not controlled. Third, MacDorman and colleagues failed to distinguish mentalizing from submentalizing explanations of their data; in reporting altercentric interference, the researchers did not analyze the extent to which reaction-times and response errors in the self-inconsistent trials for social stimuli (e.g., human avatar) were different from the non-social stimuli (e.g., arrow).

In Experiment 3, MacDorman et al. (2013) provided some degree of experimental control over the stimuli. They presented adults with the dot perspective task where the male agent in the room was presented at three levels of human photorealism. The researchers first checked that an intermediary level of human photorealism (a 3D human character rendered using FaceGen from frontal and profile photos of a man's photo) was rated as being eerier than a lower level of human photorealism (a 2D cartoonised-version of the man's face) or a higher level of human photorealism (actual photo of the man). Again, Experiment 3 failed to show any moderating influences of human photorealism or eeriness; only the general altercentric interference effect was observed (but it is important to realize that the submentalizing hypothesis still cannot be discounted as all of the different faces are similarly oriented to one side of the room or another). It is, nonetheless, challenging to interpret the negative findings. Although three kinds of pictures (i.e., photographs, two-dimensional computer models and three-dimensional computer models) were made based on the same characters were divided into three levels of eeriness (low, medium, high), whether these three levels have statistically significant differences and whether the eeriness of 3D computer models are high enough for the uncanny valley to interfere with potential implicit mentalizing are still unknown. To address these issues, future studies should use effective independent variable manipulation and effectively measure dependent variables to ensure that an eeriness level is sufficiently high to elicit the UV effect, rather than ratings animacy and eeriness after the dot-perspective task in the prior research (MacDorman et al., 2013). Based on findings of a meta-analysis (Diel et al., 2021), face distortion may be considered for stimulus creation because it produces a larger effect size of UV effect than other independent variable operationalizations; instead of measuring the eeriness of characters after the dot-perspective task (MacDorman et al., 2013), the eeriness should be tested and different eeriness levels should be statistically significant before measuring the relationship between the UV and dot-perspective task. Recently, Wahn et al.'s (2023) study supported the submentalizing account by showing triggered level-1 VPT no matter when the robot (human-like or artificial head) was switched on (i.e., mental ability for perception was available) or off (i.e., mental ability for perception was unavailable). Nevertheless, the eeriness of the robot was not measured, so whether the manipulation of the robot may elicit uncanny valley effect remains unclear. Altogether, whether the uncanny valley affects level-1 VPT and whether the research issue provide new insight into the implicit mentalizing vs. submentalizing debate remain areas of active investigation.

## 5 Open questions

In the future studies, the following issues can be explored. First, future research can focus on the extent to which facial stimuli falling into the uncanny valley interferes with automatic operations of L1VPT on Samson et al.'s (2010) dot perspective task. There will be certain improvements to the groundwork laid by MacDorman et al. (2013). First, to control varying numbers of directional cues as typically happens when different kinds of characters are used and to make sure of the ecological validity of the characters (being capable of mindreading), the kind of stimuli used for the dot perspective task will be smiling face images of real human beings and their manipulations. Second, representations of faces (actual and those resembling a human face with vestigial nonhuman features) will be created according to realism inconsistency theory; all face stimuli will be rated beforehand on humanness, warmth, eeriness and attractiveness indices [developed by Ho and MacDorman (2010, 2017)] to ensure that we end up using and testing stimuli that actually dips into the uncanny valley. Additionally, studies providing support for realism inconsistency theory as a road to the UV phenomenon indicate that negative affinity evaluations may be triggered when the mismatch between the realism of the eyes and faces are the greatest (MacDorman et al., 2009; Seyama and Nagayama, 2007). Therefore, we can manipulate eyes and faces when we intend to create eerie faces. Third, studies will add clearly semi-social stimulus (i.e., 3D arrow) and non-social stimuli (i.e., 3D triangle and 2D rectangle) with features maintaining their realism as the other kinds of stimulus to distinguish mentalizing from submentalizing operations of L1VPT. Fourth, in Samson et al.'s standard procedure, they presented the instruction (“You” or “Him”/“Her”) first, then a number, and then the visual display (room with the dots and one of the characters). Here it is proposed that the instruction will be presented after the visual display to allow time for the central stimulus and its properties to be processed. This adjustment to the Samson et al.'s procedure is expected to enhance sensitivity in detection of modulation of altercentric interference in the self-trials by preventing early filtering of the character's face through selective attention (MacDorman et al., 2013). Second, the above future study will use the eerie faces around the dip of Mori's graph as our stimuli to investigate the UV effect on level-1 VPT; what we can do later is to quantify the effect. Specifically, Lay et al. (2016) proposed a principle that a full range of empirical data for replicating the Mori's graph would include 0%, 25%, 50%, and 75% human likeness, and 100% human. Thus, we can create face images with five levels of realism (0%, 25%, 50%,75%, and 100%) and probe their corresponding effects on level-1 VPT. Third, some work on UV effect is based on the realism inconsistency theory; we can examine if categorizing ambiguous entities can lead to the UV effect as Yamada et al.'s (2013) work has shown that categorically ambiguous stimuli are also uncanny (Yamada et al., 2013), and if so, whether it has the same effects on level-1 VPT. Thus, a control for realism inconsistency mechanism causing uncanniness should be included in addition to the manipulation of ambiguity of the stimuli. Specifically, similar to Diel and Lewis's (2022) manipulations of sentence ambiguity and deviation conditions to explore whether the uncanniness was due to either deviations from typical category members or the ambiguity of the stimuli, when creating stimuli, ambiguous stimuli without any configural distorted features (ambiguity condition), non-ambiguous counterparts without any configural distorted features (non-ambiguity condition) and non-ambiguous counterparts with configural distorted features (deviation condition) may be considered. Fourth, we may dissociate between the competing mentalizing and submentalizing accounts under the impact of UV by adopting a standard Posner cueing task (for investigation of attentional orienting), and manipulating SOA and eye gaze aversion (gaze-maintained: when head orientation is aligned with eye gaze; gaze-averted: when head and eye gaze are not aligned) as the other two factors. Gardner et al. (2018) have used the Posner detection and avatar tasks to find that gaze aversion modulates attention orienting at longer SOA but not perspective taking, and averted eye gaze have been found to generate feeling of ostracism when compared to direct eye contact (Kiilavuori et al., 2021; Wirth et al., 2010). These findings raise the possibility that eye gaze aversion may facilitate the UV effect, leading a stronger reduction on altercentric interference effect in the avatar task; eye gaze aversion may moderate attention orienting regardless of UV effect. Finally, we can compare which part, face alone, body alone, whole body or combinations of human beings' and robots' faces and bodies, contributes more to UV effect and its effect on level-1 VPT.

## 6 Conclusion of the review

Our review demonstrates that there is an implicit mentalizing vs. submentalizing debate about L1VPT and the UV effect can be explained from cognitive, perceptual and evolutionary viewpoints. Several manipulations (e.g., opaque and transparent visual barriers, task type) have been adopted to explore the underlying mechanism of L1VPT. Interestingly, the UV effect may open new avenues for addressing the debate on L1VPT. However, the relationship between the UV effect and L1VPT is still understudied, which warrants further investigation. In summary, our review can help shed light on the underpinning mechanism of L1VPT and give us a deeper understanding of the mechanism via the UV effect, which may be beneficial for AI development.
